# Commentary on *JPOSNA®* paper, “Dermatitis after spine fusion caused by liquid adhesive (2-octyl cyanoacrylate)”

**DOI:** 10.1016/j.jposna.2024.100044

**Published:** 2024-04-05

**Authors:** Lisa M. Arkin, Margo Reeder

**Affiliations:** Department of Dermatology, University of Wisconsin School of Medicine and Public Health, Madison, WI, USA

Liquid 2-octyl cyanoacrylate (Derma-Bond) is a UV curing skin adhesive that can be implemented for a variety of wound closures including in spinal surgery. Acrylates rapidly polymerize into solid chains, leading to rapid, durable wound closure, without the need for dedicated removal required of sutures and staples. However, cyanoacrylates can trigger allergic contact dermatitis (ACD), complicating recovery from surgery. In this May 2024 issue of *JPOSNA®*, Miller et al. report 2 cases of ACD from 2-octyl cyanoacrylate adhesive. This paper highlights an uncommon but treatable complication following surgical repair using liquid adhesives. Early recognition of ACD and treatment are critical aspects of this Quality Improvement Case Series report for the pediatric orthopaedic community.

ACD is a delayed, T-cell mediated hypersensitivity reaction that will recur with each subsequent exposure. Studies reporting ACD from the use of liquid adhesives containing acrylates in orthopaedic surgery collectively report an incidence of 0.5% to 2.7% [1], [2], [3], suggesting that alternative wound closures may not be necessary for most patients given the benefits reported for liquid adhesive closures in orthopaedic surgery. ACD typically manifests with well demarcated, pruritic patches and vesiculated plaques that align with the application of the liquid skin adhesive and usually occurs within the first 2 weeks after exposure. Importantly, many patients with ACD to 2-octyl cyanoacrylate are not previously sensitized and may develop ACD after the first exposure, reacting up to 30 days after exposure [4]. ACD to 2-octyl cyanoacrylate could be confused with an early would infection, but the prominent itching suggests allergy over infection. In uncertain cases, culture can be helpful early on, but persistent dermatitis can also lead to secondary infection due to a broken skin barrier.

Most cases of Dermabond ACD should be relatively straightforward to diagnose, but if in question patch testing may be done to confirm allergy. Patch testing with the TRUE system will miss ACD to 2-octyl cyanoacrylate so expanded testing must be performed. However, even patch testing with ethyl cyanoacrylate can also miss allergy from Dermabond, as only 20% of the patients allergic to 2-octyl cyanoacrylate in Dermabond are positive to ethyl cyanoacrylate [5]. Diagnostic yield is increased if patch testing is done directly to 2-octyl cyanoacrylate on abraded skin.

The authors of this series propose an algorithm for treatment including use of high potency topical steroids, mupirocin ointment to the wound site, and antihistamines for pruritus. High potency topical steroids applied twice daily to the affected area are the most important part to treat both the itching and dermatitis. Topical mupirocin and antihistamines may not be necessary. We would add that earlier identification of the reaction will improve outcomes as patients with severe allergic contact dermatitis are at risk for the development of an id hypersensitivity reaction leading to the development of eczematous dermatitis at sites distant from the primary exposure (Fig. 1). This is often mistaken for a drug reaction but is an auto inflammatory response resulting from contact dermatitis. Given the severity and widespread reaction, patients with id reactions frequently require oral steroids as the authors noted in one of their patients. Because oral steroids increase the risk for poor wound healing and surgical site infections, recognition of dermatitis is critical, as earlier initiation of appropriate treatment often aborts this reaction.

**Figure 1:**
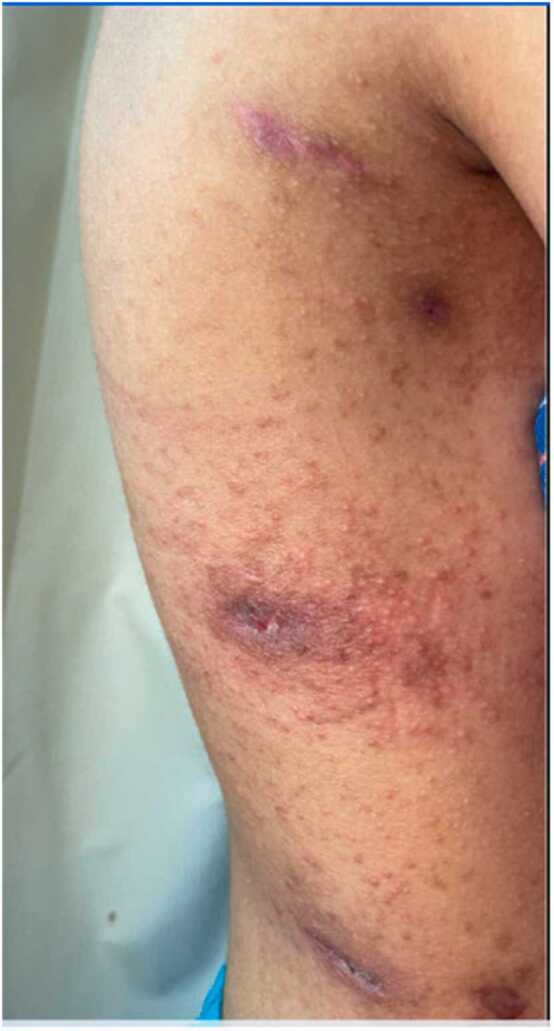
Id reaction to allergic contact dermatitis to Dermabond, with generalized pinpoint pruritic papules distant from the primary site. This reaction is often associated with delayed recognition of primary dermatitis and may necessitate the administration of oral prednisone.

ACD to 2-octyl cyanoacrylate is uncommon and for most patients should not alter the surgical strategies for spinal surgery closure. However, surgeons should maintain a high degree of suspicion for ACD if patients develop pruritic dermatitis in the areas of Dermabond application, keeping in mind that some patients may react >30 days after exposure. Informing patients that pruritus, blistering and scaly eruptions at the surgical site should prompt an early phone call to manage complications. When the diagnosis is unclear, patients with dermatitis after surgery should be sent to a dermatologist with expertise in patch testing to help orthopaedic surgeons to prevent this complication and optimize outcomes for their patients.

## Declarations of competing interests

The authors declare that they have no known competing financial interests or personal relationships that could have appeared to influence the work reported in this paper.
